# A comprehensive dosimetric study on switching from a Type-B to a Type-C dose algorithm for modern lung SBRT

**DOI:** 10.1186/s13014-017-0816-x

**Published:** 2017-05-05

**Authors:** Christina Zhou, Nathan Bennion, Rongtao Ma, Xiaoying Liang, Shuo Wang, Kristina Zvolanek, Megan Hyun, Xiaobo Li, Sumin Zhou, Weining Zhen, Chi Lin, Andrew Wahl, Dandan Zheng

**Affiliations:** 1 School of Biological Sciences, University of Chicago, Chicago, IL USA; 2 Department of Radiation Oncology, University of Nebraska Medical Center, 42nd and Emile St, Omaha, NE 68198 USA; 3 University of Florida Health Proton Therapy Institute, Jacksonville, FL USA; 4 Department of Biological Systems Engineering, University of Nebraska Lincoln, Lincoln, NE USA; 5 Department of Radiation Oncology, Fujian Medical University Union Hospital, Fuzhou, Fujian China

**Keywords:** Lung, SBRT, Dose algorithms, Monte Carlo, VMAT

## Abstract

**Background:**

Type-C dose algorithms provide more accurate dosimetry for lung SBRT treatment planning. However, because current dosimetric protocols were developed based on conventional algorithms, its applicability for the new generation algorithms needs to be determined. Previous studies on this issue used small sample sizes and reached discordant conclusions. Our study assessed dose calculation of a Type-C algorithm with current dosimetric protocols in a large patient cohort, in order to demonstrate the dosimetric impacts and necessary treatment planning steps of switching from a Type-B to a Type-C dose algorithm for lung SBRT planning.

**Methods:**

Fifty-two lung SBRT patients were included, each planned using coplanar VMAT arcs, normalized to D_95%_ = prescription dose using a Type-B algorithm. These were compared against three Type-C plans: re-calculated plans (identical plan parameters), re-normalized plans (D_95%_ = prescription dose), and re-optimized plans. Dosimetric endpoints were extracted and compared among the four plans, including RTOG dosimetric criteria: (R_100%_, R_50%_, D_2cm_, V_105%_, and lung V_20_), PTV D_min_, D_max,_ D_mean,_ V_%_ and D_90%_, PTV coverage (V_100%_), homogeneity index (HI), and Paddick conformity index (PCI).

**Results:**

Re-calculated Type-C plans resulted in decreased PTV D_min_ with a mean difference of 5.2% and increased D_max_ with a mean difference of 3.1%, similar or improved RTOG dose compliance, but compromised PTV coverage (mean D_95%_ and V_100%_ reduction of 2.5 and 8.1%, respectively). Seven plans had >5% D_95%_ reduction (maximum reduction = 16.7%), and 18 plans had >5% V_100%_ reduction (maximum reduction = 60.0%). Re-normalized Type-C plans restored target coverage, but yielded degraded plan conformity (average PCI reduction 4.0%), and RTOG dosimetric criteria deviation worsened in 11 plans, in R_50%_, D_2cm_, and R_100%_. Except for one case, re-optimized Type-C plans restored RTOG compliance achieved by the original Type-B plans, resulting in similar dosimetric values but slightly higher target dose heterogeneity (mean HI increase = 13.2%).

**Conclusions:**

Type-B SBRT lung plans considerably overestimate target coverage for some patients, necessitating Type-C re-normalization or re-optimization. Current RTOG dosimetric criteria appear to remain appropriate.

## Background

Lung cancer is the second most common type of cancer for both men and women, and the leading cause of cancer death, making up about 1 in every 4 cancer-related deaths [1]. Non-small cell lung cancer (NSCLC) makes up about 80–85% of all cases. Treatment for NSCLC varies depending on the cancer stage and other patient factors, but some common treatments include surgery, chemotherapy, immunotherapy, and radiation therapy. Stereotactic body radiotherapy (SBRT), also known as stereotactic ablative radiotherapy (SABR), is a type of external beam radiotherapy that is often used to treat early stage lung cancers as an increasingly popular alternative option to surgery [2–4]. It is also often used to treat small-sized, oligo-metastasis in the lung [5].

Lung SBRT conventionally used multiple coplanar or non-coplanar conformal beams [6]. In recent years, volumetric-modulated arc therapy (VMAT) has gained greater popularity as the treatment technique of choice for lung SBRT because of the quick delivery time, superior critical tissue sparing and dose conformity, and robustness against the interplay effect due to respiratory motion [7–12]. Several studies showed a substantial reduction in the treatment time for SBRT lung cases using VMAT when compared to intensity-modulated radiation therapy (IMRT) and conformal beam treatment, from dozens of to just a few minutes, especially when combined with the high dose rates provided by flattening-filter-free modes of modern linear accelerators (LINACs) [7, 9, 11]. While offering comparable steep dose gradients and critical tissue sparing to IMRT, VMAT was also found to be less susceptible to the interplay effect that prevented the wide application of IMRT in thoracic radiotherapy, especially when conventional dose rates are used [8, 10, 12]. However, accurate dose calculation in the presence of heterogeneity remains a challenge that arises when using SBRT [13–18]. The heterogeneous tissue interfaces between low density lung tissue and high density tumor pose challenges for accurate dose modeling. Starting from homogeneous dose calculation, commercial treatment planning systems progressed in generations of heterogeneity corrected calculation algorithms, often classified as Type-A to Type-C, to better address this challenge [19–21]. Conventional homogeneous dose calculations and Type-A or pencil beam algorithms with equivalent path length corrections can often lead to target peripheral dose over-estimation as high as 50% [22–25]. As the current mainstay of commercial treatment planning algorithms, Type-B or convolution/superposition dose algorithms are commonly used due to their improved accuracy compared with older algorithms [17, 26]. On the other hand, studies have shown that while these algorithms resulted in <3% errors on the peripheral target dose in many patient cases, errors could be as high as over 10% compared with Monte Carlo calculation in other cases [27–30]. Therefore, Type-C or fast Monte Carlo algorithms have been implemented in commercial treatment planning systems in the recent decade, which provide improved dose calculation accuracy over Type-B algorithms but faster dose modeling than full-fledged Monte Carlo simulation [29, 31–33]. Acuros^®^ XB dose calculation algorithm, implemented for treatment planning in Eclipse (Varian Medical Systems, Palo Alto, CA), is based on a linear Boltzmann equation solver to solve radiation transport equation, rather than simulation of particle transport implemented in Monte Carlo. It is considered a Type-C algorithm due to the comparable level of dose modeling accuracy [30, 34–36].

Many studies have investigated the dosimetric differences between Type-A or Type-B and Type-C dose algorithms, and have shown that Type-C algorithms more accurately calculate the dose distribution for SBRT plans of lung patients [15, 22–24, 27, 32, 35]. Due to the large dose errors associated with Type-A algorithms, they have been gradually phased out in thoracic treatment planning. Modern lung SBRT protocols from National Research Group (NRG)/Radiation Therapy Oncology Group (RTOG) such as RTOG0813 and RTOG0915 require Type-B dose calculation [37, 38]. Despite the demonstrated dose calculation accuracy improvement and the increasing availability, currently the clinical utilization of Type-C algorithms is still limited [5]. In addition to technical challenges such as lengthier computation time and sub-optimal or lack of integration into inverse optimization engines compared with Type-B algorithms, there are also some clinical challenges. Firstly, inhomogeneous dose errors were revealed for Type-B compared with Type-C algorithms for different patients. While large errors can result for some patients, small errors within 3% were found for other patients [27–30], suggesting that elaborate dose calculation using Type-C algorithms may not be necessary for every lung SBRT patient to offset the lengthier computation time. Secondly, current clinical guidelines on dose prescription and constraints have been established for Type-B algorithms and may not be directly applicable for Type-C algorithms.

Several authors have previously examined the dosimetric compliance of Type-C calculated lung SBRT plans to the current protocols [27, 29, 39, 40]. However, discordant recommendations were reported by these studies. Li et al. applied Monaco (Elekta/CMS, Crawley, UK), a Type-C algorithm, on 15 patients planned using XiO (Elekta/CMS, Crawley, UK), a Type-B algorithm, to examine RTOG 0813 compliance [29]. They recommended adjusting the dosimetric criteria (such as R_50%_) because their values were increased with the Type-C algorithm and the corresponding compliance was worse. In contrast, Rana et al. published a study on 14 patients evaluating the effect of switching from a Type-B algorithm (Analytical Anisotropic Algorithm, or AAA) to a Type-C algorithm (AXB) in Eclipse for lung SBRT plans using RTOG0813 dosimetric criteria [27]. They found that the AXB re-calculation of the original AAA plans led to lower average values for most dose constraint parameters and reduced instances of protocol minor deviations on these parameters. Two studies by Pokhrel et al. used another Type-C algorithm, Voxel Monte Carlo implemented in iPlan (BrainLab AG, Feldkirchen, Germany), to evaluate the plan compliance with dosimetric criteria of RTOG0813 and RTOG0915 for central and peripheral lung SBRT patients, respectively [39, 40]. Based on the average values obtained on 20 patients, Pokhrel et al. recommended the necessary adjustments to the dosimetric criteria in order to make their Type-C plans fully compliant with the protocols.

The aim of this study, therefore, was to systematically investigate the necessity and dosimetric impacts of switching from Type-B to Type-C dose algorithms for lung SBRT planning on a large patient cohort. On fifty-two patients, the target dose parameters as well as RTOG dosimetric criteria were compared among the following plans: original Type-B plans, re-calculated Type-C plans with identical beam settings, re-normalized Type-C plans (to ensure target coverage), and re-optimized Type-C plans (to restore the original compliance to the dosimetric criteria achieved by the Type-B plans).

## Methods

As will be detailed in the following sections, the dosimetric impacts of switching from Type-B to Type-C calculation were comprehensively evaluated using various dose-volume endpoints and protocol dosimetric constraints on 52 lung SBRT patient plans. We compared the original Type-B plans with the re-calculated, re-normalized, and re-optimized Type-C plans.

### Original Type-B plans

With the approval of the University of Nebraska Medical Center institutional review board, treatment data for 70 patients with NSCLC, treated with lung SBRT at our institution between April 2014 and August 2016, were collected. From these, 52 plans treated with VMAT were selected for this retrospective study. The remaining cases were excluded because they used 3D conformal treatment techniques.

According to our institutional protocol, each patient received a free-breathing three-dimensional computed tomography (3D CT) followed by an eight-phase four-dimensional computed tomography (4D CT) acquired using a Sensation Open CT simulator (Siemens, Erlangen, Germany) and the Real-time Position Management system (Varian Medical Systems, Palo Alto, CA) as the respiratory surrogate. A slice thickness of 2 mm was used for both CT scans. Patients were simulated in treatment position and immobilized with the BlueBAG™ immobilization system (Medical Intelligence, Schwabmünchen, Germany). The 3D and 4D CT images were fused using the common frame of reference if visually confirmed with no relative movement, or otherwise using rigid registration to the spine. For each patient, the gross tumor volume (GTV) was delineated by the attending radiation oncologists as the visible tumor on the 3D CT, and the internal target volume (ITV) was contoured using the 3DCT, maximum intensity projection (MIP) from the 4DCT, and each phase of the 4DCT, as the union of the gross tumor seen on these images excluding possible nearby soft-tissue. The planning target volume (PTV) was generated by adding an isotropic expansion of 5–6 mm to the ITV.

For treatment planning, one to two coplanar VMAT arcs were used, with partial arcs on the ipsilateral side to spare the contralateral lung for some peripheral lesions and full arcs for the rest. Fifty plans used flattening-filter-free 6 MV and/or 10 MV beams, and the rest used flattened beams of these energies. The plans were optimized to best comply with the dosimetric criteria and normal tissue constraints specified in RTOG0813, RTOG0915, other guidelines [37, 38, 41], and according to our institutional protocol. On average, the prescription dose was about 88% of the maximum dose, with the lowest prescription isodose line at 76% of the maximum dose for one plan. All the treatment plans specified a dose prescription of either 48 Gy (12 Gy x 4 fractions) or 50 Gy (10 Gy x 5 fractions). Additionally, all cases were planned using Type-B algorithms, with 47 plans using Analytical Anisotropic Algorithm, or AAA in Eclipse 13.5 (Varian Medical Systems, Palo Alto, CA) and 5 plans using collapsed cone convolution, or CCC in Pinnacle [3] (Philips Medical, Fitchburg WI). Plans originally planned in Pinnacle [3] were transferred into Eclipse and re-calculated with AAA for this study. All plans were normalized so that 95% of the PTV received the full prescription dose (D_95%_ = prescription dose).

### Re-calculated Type-C plans

The above plans were re-calculated using a Type-C algorithm, Acuros XB or AXB in Eclipse 13.5. Identical beam parameters including MLC patterns and monitor units were used for the re-calculation as in the original Type-B, or AAA plans. For AXB calculations, dose-to-medium was reported.

### Re-normalized Type-C plans

Another set of treatment plans was generated by re-calculating the original clinical treatment plans with the Type-C algorithm (AXB), but specifying plan normalization so that 95% of the target volume received the full prescription dose (D_95%_ = prescription dose) as in the original clinical Type-B plans. In other words, these plans used identical MLC patterns and relative beam weighting as the original plans, but with different MUs so that the Type-C plans’ PTV doses were re-normalized to match the original Type-B, or AAA plans (D_95%_ = prescription dose).

### Re-optimized Type-C plans

For the re-normalized Type-C plans that did not meet the level of RTOG dosimetric compliance seen in their respective original Type-B plans, re-optimization was performed using the Type-C algorithm, keeping the normalization at D_95%_ = prescription dose, to improve the dosimetric compliance. Specifically, in addition to dose objectives for critical organs, the following dosimetric criteria from the protocols were utilized: ratio of prescription isodose volumes to PTV (R_100%),_ ratio of 50% prescription isodose volume to PTV (R_50%_), maximal dose 2 cm from the PTV in any direction as a percentage of prescription dose (D_2cm_), the percentage of lung receiving dose equal to or larger than 20 Gy (V_20_), and the volume of 105% isodose outside the PTV (V_105%_). The optimization objectives were adjusted to create plans that optimize these dosimetric values with intermediate and final Type-C dose calculation.

### Dosimetric evaluation and comparison

For all above plans, the minimum (D_min_), maximum (D_max_), and mean (D_mean_) doses of the PTV were recorded, along with the doses received by 95% (D_95%_) and 90% (D_90%_) of the PTV volume. Volume-based dosimetric parameters such as D_95%_ were included and used for plan normalization because single-point-based doses such as D_min_ are associated with higher uncertainties. The PTV coverage, referring to the percent volume of PTV covered by prescription dose or V_100%_, was also recorded. RTOG criteria were used to calculate the values for R_100%_, R_50%_, D_2cm_, V_20_, and V_105%_. Additionally, the Paddick Conformity Index (PCI) [42] and the homogeneity index (HI) [43] were calculated. The PCI was defined as (TV_PIV_)^2^/(TV x PIV), with TV_PIV_ being the PTV volume receiving full prescription dose, TV being the total PTV volume, and PIV being the total irradiated volume receiving full prescription dose. For any plan, the PCI would be within the range of 0 to 1.0, with PCI = 1.0 for an ideal plan. The HI was defined as the ratio of the maximum dose over the prescription dose. The above dosimetric parameters were used to compare the four types of plans as described in II.A.-II.D. for each of the 52 patients.

## Results

### Clinical characteristics

Patient and tumor characteristics of the 52 patients are described in Table 1.

**Table 1:** Patient and tumor characteristics

Parameter	Total
Patients (*n* = 52)	Female = 26, male = 26
Median age in years (range)	73 (46–89)
Median PTV in cm^3^ (range)	22.4 (6.1–85.7)
Tumor location (*n* = 52)	13 LUL, 14 RUL, 7 LLL, 14 RLL, 4 RML

*LUL* left upper lobe; *RUL* right upper lobe; *LLL* left lower lobe; *RLL* right lower lobe; *RML* right middle lobe

### Re-calculated and re-normalized Type-C plans vs. original Type-B plans

For the 52 patients, the average values and standard deviations are summarized in Table 2 for PTV dosimetric parameters, and in Table 3 for PTV coverage (V_100%_), PCI, and HI. The numbers of cases with deviations to RTOG dosimetric criteria are listed in Table 4. These were used to compare between re-calculated Type-C plans, re-normalized Type-C plans, and the original Type-B plans.

**Table 2:** PTV dosimetric data averages and standard deviations over all patients for the original Type-B plans and the re-calculated as well as re-normalized Type-C comparison plans

	D_min_ (Gy)	D_mean_ (Gy)	D_max_ (Gy)	D_95%_ (Gy)	D_90%_ (Gy)
Original Type-B	45.5 ± 2.6	52.5 ± 1.6	56.5 ± 3.8	49.5 ± 0.9	50.1 ± 1.0
Re-calculated Type-C	43.2 ± 3.4	52.5 ± 2.3	59.6 ± 3.4	48.4 ± 2.9	49.3 ± 2.4
Re-normalized Type-C	44.1 ± 2.4	53.7 ± 1.9	59.6 ± 3.9	49.5 ± 0.9	50.5 ± 0.9

**Table 3:** PTV coverage (defined as V_100%_), Paddick conformity index (PCI), and homogeneity index (HI) averages and standard deviations over all patients for the original Type-B plans and re-calculated as well as re-normalized Type-C comparison plans

	PTV Coverage (V_100%_) (%)	PCI	HI
Original Type-B	92.2 ± 3.3	0.9 ± 0.1	1.1 ± 0.1
Re-calculated Type-C	84.8 ± 14.6	0.8 ± 0.1	1.2 ± 0.1
Re-normalized Type-C	92.7 ± 2.5	0.8 ± 0.1	1.2 ± 0.1

**Table 4:** RTOG criteria compliance (numbers of cases with deviations) for the original Type-B plans and re-calculated as well as re-normalized Type-C comparison plans

	R_100%_	R_50%_	D_2cm_
Original Type-B	0 deviation	30 minor deviations	16 minor deviations
Re-calculated Type-C	0 deviation	27 minor deviations	11 minor deviations
Re-normalized Type-C	3 minor deviations	29 minor deviations, 5 major deviations	16 minor deviations, 1 major deviation

**Fig. 1:**
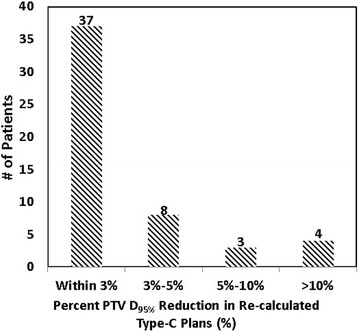
Distribution of the magnitude for PTV D_95%_ reduction on re-calculated Type-C plans

**Fig. 2:**
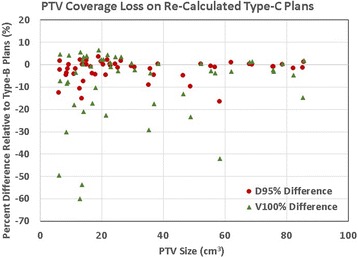
PTV coverage loss (as in D_95%_ and V_100%_ reductions) on re-calculated Type-C plans over PTV size

As shown in Table 4, re-calculated Type-C plans resulted in comparable or improved compliance for RTOG dose criteria compared with the original Type-B plans. Of the original 52 Type-B plans, 30 plans had minor deviations for R_50%_ and 16 had minor deviations for D_2cm_. None had deviations for R_100%_, V_20_, or V_105%_, and no major deviations were observed for any of the criteria. After re-calculating with Type-C algorithm, the level of deviation was reduced from minor to none for 3 plans for R_50%_ and similarly in 5 plans for D_2cm_. On the other hand, these re-calculated Type-C plans revealed the target coverage dose overestimation by the Type-B algorithm, as can be seen in the reduced PTV D_min_, D_95%_, and D_90%_ in Table 2, and in the reduced PTV coverage or V_100%_ in Table 3. It is worth noting that although the 2.3% average PTV D_95%_ reduction may not seem clinically significant, a wide range of variation (standard deviation = 4.4%) was observed from patient to patient, with a maximum reduction of 16.7%. Of the 52 patients, 7 patients (13.5%) showed PTV D_95%_ a reduction over 5%, and 4 patients (7.7%) showed a reduction over 10%. Figure 1 plots the distribution of the magnitude for PTV D_95%_ reduction. Similarly, PTV V_100%_ was reduced on average by 7.4% from 92.2 ± 3.3% to 84.8 ± 14.6%. Of the 52 patients, 18 patients (34.6%) showed a PTV V_100%_ reduction over 5%, and 15 patients (28.8%) showed a reduction over 10%. The maximum V_100%_ reduction was 60% for one patient. Figure 2 plots the PTV D_95%_ and V_100%_ reductions for all patients over PTV size.

The PTV coverage reductions on the re-calculated Type-C plans suggested that simply re-calculating the plans with the Type-C algorithm might not provide fair comparisons. For example, on the 8 plans with improved RTOG dose compliance on the re-calculated Type-C plans when compared with the original Type-B plans, the average PTV D_95%_ reduction was 4.2%, with a maximum reduction of 15.1%. We therefore designed re-normalized Type-C plans to restore the dose coverage by ensuring D_95%_ = prescription dose through inflated MUs. Not surprisingly, the average values for the RTOG dosimetric criteria increased on the re-normalized plans for R_100%_, R_50%_, and D_2cm_ compared with the original Type-B plans. On 11 out of the 52 plans (21.2%), protocol compliance degraded on one or more dosimetric criteria when compared with the original Type-B plans. For R_50%_, 5 plans changed from minor to major deviation, and 4 plans from no deviation to minor deviation. For D_2cm_, 1 plan changed from minor to major deviation, 6 plans from no deviation to minor deviation, and 5 plans from minor deviation to no deviation. For R_100%_, 3 plans changed from no deviation to minor deviation. No plans showed changes for V_105%_ or lung V_20_.

**Fig. 3:**
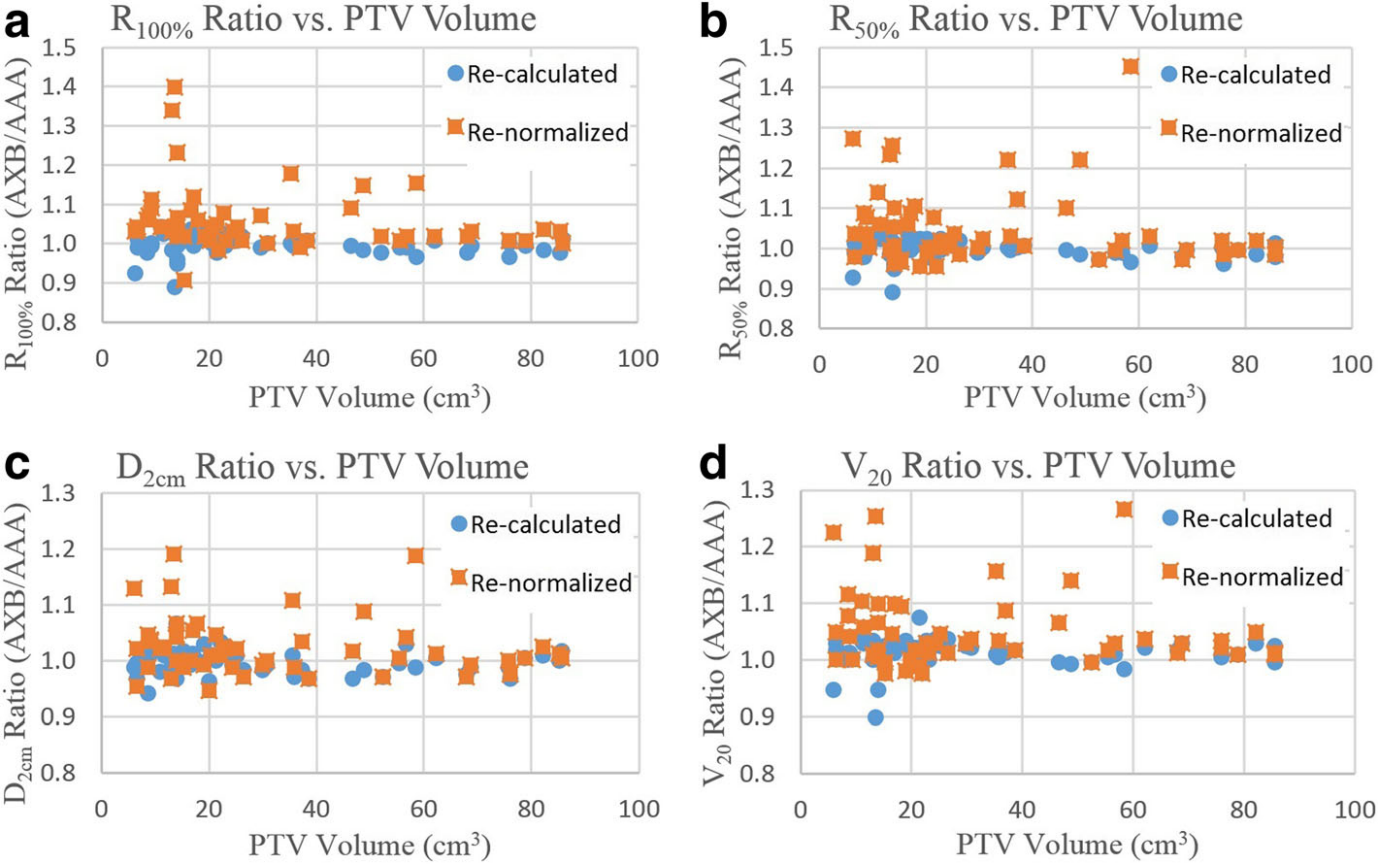
The ratios of the re-calculated and re-normalized Type-C plans over the original Type-B plans are plotted against PTV volume for RTOG dosimetric parameters (**a**) R_100%_, (**b**) R_50%_, (**c**) D_2cm_, and (**d**) lung V_20_

The ratios of the re-calculated and re-normalized Type-C plans over the original Type-B plans are plotted against PTV volume in Fig. 3(a)-(d) for RTOG dosimetric parameters R_100%_, R_50%_, D_2cm_, and lung V_20_, respectively. It is apparent that the ratios for the re-calculated plans are more clustered around or below 1.0 and those for the re-normalized plans are more spread out and above 1.0. This indicates that while re-calculated Type-C plans achieved similar or even better RTOG dosimetric criteria compliance as the original Type-B plans, the compliance is worsened on the re-normalized Type-C plans in which adequate target dose coverage was restored.

### Re-optimized Type-C plans

For the 11 patients whose re-normalized Type-C plans showed worsened RTOG dosimetric criteria compliance for R_50%_, D_2cm_ and/or R_100%_, re-optimization was performed using the Type-C algorithm. While keeping the original beam arrangements and collimator angles, new optimization objectives were necessary for Type-C optimization, different from those used for the original Type-B optimization. On these 11 patients, the ratios of the re-optimized Type-C plan over the original Type-B plan were listed for PTV dosimetric parameters, RTOG criteria, and other plan quality indexes in Tables 5, 6, and 7, respectively. The PTV volumes and the PTV D_95%_ reduction revealed by the re-calculated plans are also listed alongside as a reference. The original levels of compliance on the original Type-B plans were restored by the re-optimization for all but one patient, Patient #11, who continued to have a minor R_50%_ deviation after re-optimization while the original Type-B plan had no deviation.

**Table 5:** PTV dosimetric parameter ratios of the re-optimized Type-C plan over the original Type-B plan for the 11 re-optimized patients

Pt #	PTV Volume (cm^3^)	D_95%_ Reduction (%)	Ratio D_min_	Ratio D_mean_	Ratio D_max_	Ratio D_95%_	Ratio D_90%_
1	6.1	12.5	0.9	1.1	1.2	1.0	1.0
2	6.5	2.3	0.9	1.1	1.2	1.0	1.0
3	13.0	10.6	1.0	1.1	1.1	1.0	1.0
4	13.6	15.1	0.9	1.1	1.1	1.0	1.0
5	14.1	7.4	1.0	1.0	1.1	1.0	1.0
6	17.0	4.1	1.0	1.1	1.2	1.0	1.0
7	18.0	4.5	1.0	1.0	1.0	1.0	1.0
8	35.4	9.1	1.0	1.0	1.0	1.0	1.0
9	37.1	4.7	1.0	1.0	1.0	1.0	1.0
10	48.8	9.7	0.9	1.2	1.4	1.0	1.0
11	58.4	16.7	1.0	1.1	1.1	1.0	1.0

Also listed are the PTV volumes and PTV D_95%_ reduction revealed by the corresponding re-calculated Type-C plans as a reference. The patients are sorted by the PTV size

**Table 6:** RTOG dosimetric parameter ratios of the re-optimized Type-C plan over the original Type-B plan for the 11 re-optimized patients

Pt #	PTV Volume (cm^3^)	D_95%_ Reduction (%)	Ratio R_100%_	Ratio R_50%_	Ratio D_2cm_	Ratio V_20_
1	6.1	12.5	1.0	1.2	1.1	1.2
2	6.5	2.3	1.0	0.8	1.0	0.8
3	13.0	10.6	0.9	0.9	1.0	1.0
4	13.6	15.1	1.0	0.9	1.0	1.0
5	14.1	7.4	0.9	0.8	1.0	0.9
6	17.0	4.1	1.0	0.8	1.0	0.9
7	18.0	4.5	1.0	1.1	1.0	1.2
8	35.4	9.1	1.0	1.1	0.9	1.2
9	37.1	4.7	0.9	1.0	1.0	1.0
10	48.8	9.7	1.0	1.0	1.0	1.0
11	58.4	16.7	1.0	1.2^a^	1.0	1.2

Also listed are the PTV volumes and PTV D_95%_ reduction revealed by the corresponding re-calculated Type-C plans as a reference. The patients are sorted by the PTV size

^a^Patient# 11 had a minor deviation on R_50%_ even after Type-C re-optimization, while the corresponding original Type-B plan was fully compliant

**Table 7:** Plan quality ratios of the re-optimized Type-C plan over the original Type-B plan for the 11 re-optimized patients on PTV coverage (V_100%_), Paddick conformity index (PCI) and homogeneity index (HI)

Pt #	PTV Volume (cm^3^)	D_95%_ Reduction (%)	Ratio V_100%_	Ratio PCI	Ratio HI
1	6.1	12.5	1.0	1.0	1.2
2	6.5	2.3	1.0	1.0	1.2
3	13.0	10.6	1.0	1.0	1.1
4	13.6	15.1	1.0	1.0	1.1
5	14.1	7.4	1.0	1.0	1.1
6	17.0	4.1	1.0	1.0	1.2
7	18.0	4.5	1.0	1.0	1.0
8	35.4	9.1	1.0	1.0	1.0
9	37.1	4.7	1.0	1.1	1.0
10	48.8	9.7	1.0	1.0	1.4
11	58.4	16.7	1.0	1.0	1.1

Also listed are the PTV volumes and PTV D_95%_ reduction revealed by the corresponding re-calculated Type-C plans

**Fig. 4:**
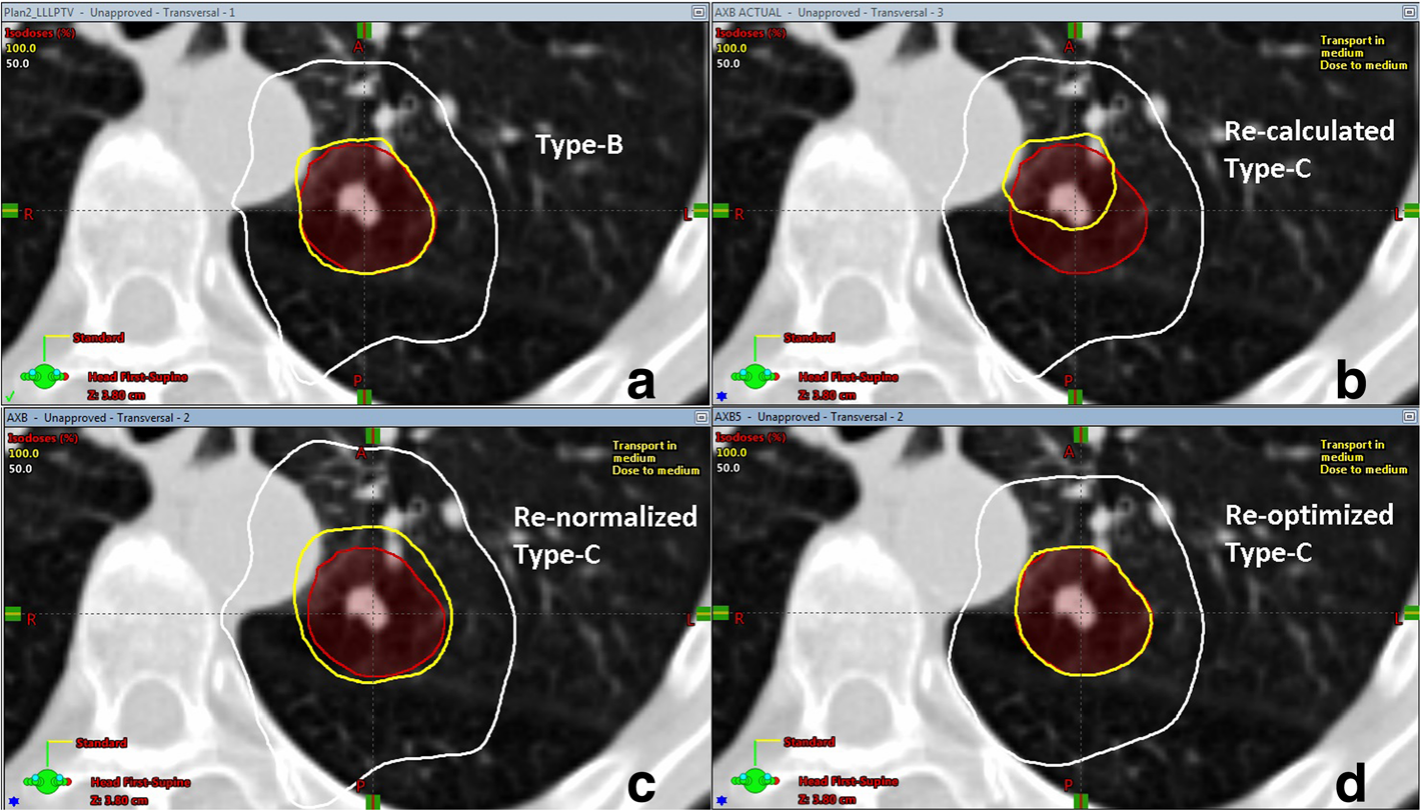
Axial isodose distributions at the isocenter for an example patient (Patient 3 in Tables 5, 6, and 7) comparing (**a**) the original Type-B plan, (**b**) the re-calculated Type-C plan, (**c**) the re-normalized Type-C plan, and (**d**) the re-optimized Type-C plan. The PTV is outlined in *red* colorwash, the 100% isodose line is marked in *yellow*, and the 50% isodose line is marked in *white*

As an example of comparing the 4 plans of the same patient, the isodose distributions in the axial view at the isocenter for Patient #3 (in Tables 5, 6, and 7) are placed side by side in Fig. 4. Compared with the original Type-B plan (a), the PTV underdose on the re-calculated Type-C plan (b) is apparent. While the re-normalized Type-C plan (c) restored the target dose coverage, the target conformity was compromised, leading to worsened R_100%_, R_50%_, and D_2cm_ values. As a result, R_100%_ deviation increased from no deviation to minor deviation, R_50%_ deviation increased from minor to major deviation, and D_2cm_ deviation increased from minor to major deviation, which necessitated re-optimization using the Type-C algorithm. On the re-optimized Type-C plan, the target conformity was restored, and the compliance to the relevant RTOG criteria was also restored to the levels previously achieved by the original Type-B plan.

## Discussion

Our study investigated target dose, RTOG compliance, and other plan quality indexes on 52 lung SBRT patients by comparing between the original Type-B plan, the re-calculated Type-C plan, the re-normalized Type-C plan, and the re-optimized Type-C plan. Although previous studies in the literature have examined dosimetric differences arising from re-calculating Type-B treatment plans with the Type-C algorithm, discordant recommendations have been made regarding the applicability of current RTOG dosimetric criteria on Type-C calculated lung SBRT plans. Specifically, the study by Li et al. using Monaco [29] and the studies by Pokhrel et al. using Voxel Monte Carlo [39, 40] suggested that the RTOG dosimetric criteria, such as R_100%_, R_50%_, and D_2cm_, might need to be up-adjusted to be more loose. In contrast, the study by Rana et al. using AXB showed comparable or even better compliance for the above criteria [27].

Our study shed some light on the conflicting results above. Similar to Rana et al.’s study [27], the re-calculated Type-C plans also showed comparable or even better compliance to RTOG dosimetric criteria, such as R_100%_, R_50%_, and D_2cm_. However, what their study failed to consider was that Type-C re-calculation using the same MLC patterns and MUs would also lead to insufficient target dose coverage and hence sub-optimal plan quality for many patients. In fact, about 13.5% of our patient cohort showed a PTV D_95%_ reduction over 5%, and about 34.6% showed a PTV V_100%_ reduction over 5%. When the target coverage loss was made up by re-normalizing the MUs, not surprisingly, compliance to the above RTOG dosimetric criteria worsened for some patients (about 21.2% of our cohort) compared with the original Type-B plans. However, re-optimization using the Type-C algorithm was able to restore the original RTOG compliance for all but one of these patients. Current RTOG dosimetric criteria hence appear to remain appropriate for lung SBRT plans calculated with Type-C algorithms, provided that such algorithms are employed for plan optimization.

Pokhrel et al. recommended up-adjustments on the values for RTOG dosimetric criteria, such as R_100%_, R_50%_, and D_2cm_, by about 10% in order for their Type-C plans to comply with the criteria [39, 40]. However, this was based on the premise that all plans need to fully comply with the criteria. Since in their studies the patients were planned with the Type-C algorithm, and no comparison was made with Type-B plans, it was not considered that for some of these 20 patients, RTOG criteria deviations could have resulted even if planned using Type-B algorithms. In fact, it is not uncommon in current clinical practice for Type-B lung SBRT plans to have minor or even major deviations from these RTOG protocols due to specific patient and tumor anatomy. For example, in the study by Rana et al., among the 14 clinical Type-B plans, 6 plans recorded minor deviations for R_100%_, 9 plans recorded minor deviations for R_50%_, 4 plans recorded minor deviations for D_2cm_, and 1 plan recorded minor deviation for V_20_. Similarly, in Li et al.’s series of 15 patients, Type-B plans also resulted in deviations on these RTOG criteria for some of them, such as the 5 minor deviations and 2 major deviations on D_2cm_ [29].

By designing a comprehensive comparison between original Type-B plan, the re-calculated Type-C plan, the re-normalized Type-C plan, and the re-optimized Type-C plan, we have clearly outlined in our study the dosimetric necessity as well as impact of re-calculation, re-normalization, and re-optimization using Type-C algorithms. While Li et al.’s study also considered re-optimization using the Type-C algorithm, the conventional conformal beam treatment technique was used in their study, and their re-optimization involved only adjusting the relative beam weights while keeping all other beam parameters the same as the original Type-B plans [29]. Furthermore, we performed our investigation on a much larger patient cohort compared with the above three studies.

Of our 52 patients, 11 (21.2%) resulted in worsened RTOG dosimetric criteria compliance on the re-normalized Type-C plans and hence necessitated re-optimization with the Type-C algorithm. These patients included all 7 patients with a PTV D_95%_ reduction over 5%. For the remaining 4 patients, 3 had a PTV D_95%_ reduction over 4% and 1 had a reduction of 2%. This indicates that for those patients on whom Type-B algorithms will lead to large PTV coverage dose overestimations, optimization by Type-C algorithms will likely be necessary to achieve similar levels of RTOG dosimetric criteria compliance currently achieved by Type-B algorithms. For patients on whom Type-B algorithms will lead to small PTV coverage dose overestimations, plans optimized with Type-B algorithms will likely only need to be re-normalized with Type-C calculation to ensure sufficient and accurate target dose coverage, as well as to achieve similar levels of RTOG compliance. In a small number of these patients, if the RTOG compliance of the Type-B plans are borderline, optimization with Type-C algorithms may also be necessary to achieve the same compliance goal.

For one patient, re-optimization using the Type-C algorithm was unable to fully restore the RTOG dosimetric criteria compliance (Patient #11 in Tables 5, 6, and 7). For this patient, the original plan was fully compliant, and the re-normalized plan resulted in minor deviation in both R_50%_ and D_2cm_. The re-optimized plan restored D_2cm_ compliance to no deviation, but on R_50%_ the re-optimized Type-C plan still had a minor deviation. Among our cohort, this patient was also the one with the highest magnitude of PTV D_95%_ reduction on the re-calculated Type-C plan, with a reduction of 16.7%. This large discrepancy in target dose calculation between Type-C and Type-B algorithms might have partially contributed to the difficulty of re-optimizing a fully-compliant plan with the Type-C algorithm. In addition, the R_50%_ value of the original Type-B plan for this patient, 3.4, was somewhat close to the minor deviation threshold of 3.8. The re-optimized plan had a value of 4.0, and hence scored as a minor deviation.

For all other patients, re-normalization or re-optimization with the Type-C algorithm achieved similar levels of RTOG compliance for these plans and notably higher target dose heterogeneity as seen in the higher HI values. The higher HI might result partially from the fact that the Type-B algorithm overestimates target peripheral dose but underestimates maximum target dose relative to the Type-C algorithm, and partially from the fact that target dose homogeneity was not heavily constrained during re-optimization. Since the current RTOG protocols recommend a wide range of prescription isodose levels from 60 to 90%, the HI values on the Type-C plans for all patients fell within this range.

Since their introductions into commercial treatment planning systems, many clinical studies have been conducted to compare dose calculation of Type-C algorithms to algorithms of previous generations [22, 23, 25, 31, 44–47]. However, their utilization in clinic practice is still very limited [5]. Two factors contributing to the lack of clinical utilization that motivated our study were: (1) the large patient-to-patient variation of error magnitudes for the current algorithm left it unclear if it is necessary to clinically switch the algorithm at the cost of increased computational time. (2) the four existing studies on the applicability of current dosimetric guidelines for Type-C algorithms had apparent disagreements in their findings [27, 29, 39, 40]. On the first issue, our study using a large patient cohort provided distribution data of target dose errors associated with Type-B calculation for different patients. Clinicians may use these data to assist making decision whether the algorithm switching is necessary. On the second issue, our study resolved the paradox from the existing literature by demonstrating the difference between re-calculated and re-normalized Type-C plans. In addition, the results on re-optimized plans shed additional light on the Type-C applicability of current RTOG guidelines, and further provided clinicians with information on different levels of Type-C involvement in treatment planning necessary to satisfy the dosimetric constraints.

One limitation of our work was that, although extensively comparing the original Type-B and re-calculated, re-normalized and re-optimized Type-C plans on a large patient cohort and with a rich pool of important dosimetric endpoints, our comparison was conducted using one Type-B algorithm, AAA, and one Type-C algorithm, AXB. In particular, although usually considered as a Type-B algorithm and currently used as a major clinical treatment planning algorithm [19–21, 28, 48–50], AAA has been shown to perform less accurately in heterogeneous environment than some other Type-B algorithms such as CCC [18, 28, 30]. However, due to the limitations of the treatment planning system, namely Pinnacle does not allow importing plans from other treatment planning systems for re-calculation and it also does not have a Type-C algorithm, a comparison to additional Type-B and Type-C algorithms could not be performed on our cohort.

It would be an interesting direction for future studies to assess the clinical impact of switching from Type-B to Type-C algorithms for lung SBRT. For example, on a cohort of patients treated with Type-B plans, the local failure rates can be compared between those patients with large PTV D_95%_ or V_100%_ reductions upon Type-C re-calculation and those patients with small reductions. This way the clinical significance of the dosimetric differences can be determined. However, due to the relatively small percentage of patients with large dose differences between Type-B and Type-C calculations (for example, in our cohort, 13.5% with a PTV D_95%_ reduction over 5%, or 34.6% with a PTV V_100%_ reduction over 5%) and the very high local control rates usually at around 90%, a very large cohort with sufficient longitudinal follow-up would be necessary to power such a study and illustrate the clinical significance of the dosimetric effects.

## Conclusions

Type-B dose calculation considerably overestimates target dose coverage in lung SBRT for some patients, necessitating Type-C re-normalization or re-optimization. Current RTOG dosimetric criteria appear to remain appropriate in the setting of Type-C calculations.

## Abbreviations

3D CT: Three-dimensional computed tomography
4D CT: Four-dimensional computed tomography
AAA: Analytical Anisotropic Algorithm
AXB: Acuros XB
GTV: Gross tumor volume
HI: Homogeneity index
IMRT: Intensity-modulated radiation therapy
ITV: Internal target volume
LINAC: Linear accelerator
MIP: Maximum intensity projection
NRG/RTOG: National Research Group/Radiation Therapy Oncology Group
NSCLC: Non-small cell lung cancer
PCI: Paddick Conformity Index
PTV: Planning target volume
SABR: Stereotactic ablative radiotherapy
SBRT: Stereotactic body radiotherapy
VMAT: Volumetric-modulated arc therapy
